# Mini-temporal approach as an alternative to the classical pterional approach for resective temporal region surgeries

**DOI:** 10.1186/s41016-022-00280-6

**Published:** 2022-09-22

**Authors:** Nijiati Kudulaiti, Feili Liu, N. U. Farrukh Hameed, Peng Wang, Jie Zhang, Rui Feng, Jinsong Wu

**Affiliations:** 1 Department of Neurosurgery, Huashan Hospital, Shanghai Medical College, Fudan University, Shanghai, China; 2 Neurosurgical Institute of Fudan University, Shanghai, China; 3 Shanghai Clinical Medical Center of Neurosurgery, Shanghai, China; 4 Shanghai Key Laboratory of Brain Function Restoration and Neural Regeneration, Shanghai, China

**Keywords:** Mini-temporal approach, Pterional craniotomy, Facial nerve, Minimally invasive surgery, Neurosurgical technique

## Abstract

**Background:**

Classical pterional appoach for temporal surgeries may cause atrophy and dysfunction of temporalis, injury to the facial nerve, and unnecessary cortical exposure. As an alternative to the classical pterional approach for such surgeries, we hereby describe an mini-temporal approach which reduces these risks and proven to be practical in neurological surgeries.

**Material and methods:**

In the mini-temporal incision design, the frontal end of the incision never surpassed the hairline at the level of temporal line, and a one-layer skin-galea-muscle flap was detached from the cranium, effectively avoiding the injuries of facial nerve. The surgical bone window was completely located underneath the temporalis muscle, allowing it to be completely repositioned postoperatively.

**Results:**

We demonstrated the application of mini-temporal approach in a variety of temporal region tumors, which can be applied to complete successful resective surgeries while effectively reducing injuries to extra-temporal cortex, temporalis, and facial nerve. There were no postoperative complications related to extra-temporal cortical damage, atrophy of temporalis, or injury to the facial nerve.

**Conclusion:**

The mini-temporal approach can effectively shorten the time of craniotomy and closure, decrease the size of bony removal, increase the restoration of temporalis during closure, and lower the chance of facial nerve injury. Therefore, it improves cosmetic outcomes and reduces the risk of unintentional extra-temporal cortical injury, which fully embodies the minimally invasive principle in neurosurgery.

## Background

Pterional craniotomy has become a mainstream surgical approach and standard in evaluating alternative surgical techniques for the same anatomical target for many years. It allows surgical exposure that minimizes brain retraction of the arterial circle of Willis, midbrain, temporal lobe, frontal lobe, supra- and parasellar regions, the orbit, cavernous sinus, and the superior orbital fissure [1]. However, due to the complete dissection of the temporal muscle, pterional craniotomy may lead to functional impairment and esthetics. Damage to the temporal branch of the facial nerve and atrophy of the subcutaneous fat pad and the temporalis often result in craniofacial contour changes. It is an inevitable trend for neurosurgery to move away from the larger craniotomies to the smaller craniotomies. As an alternative, the mini-temporal approach has been practiced in our department for many more than 8 years and achieved good results in multiple aspects. This approach fully is a practical and acceptable minimally invasive approach which demonstrates the minimally invasive principle in neurosurgery; therefore, we want to introduce the operation process and advantages of this approach to you through this technical note.

## Methods

### Operative technique

The indications of the mini-temporal approach encompass epilepsy surgeries such as anterior temporal lobectomy and selective amygdalo-hippocampectomy, glioma surgeries involving mesial and lateral temporal lobe, extra-axial tumors located in the anterior temporal region or sylvian fissure, and small tumors in the middle cranial fossa. This approach should be carefully considered in patients with hemorrhage brain tumor stroke, extremely high intracranial pressure, and the need for decompression. When designing individual incision, we designed the incision as small as possible on the premise that the tumor could be safely removed according to the preoperative navigation plan. We explained surgical procedures and risks to patients and obtained informed consent. At the same time, informed consent for academic communication and publication was obtained from all individual participants included in the study.

**Fig. 1:**
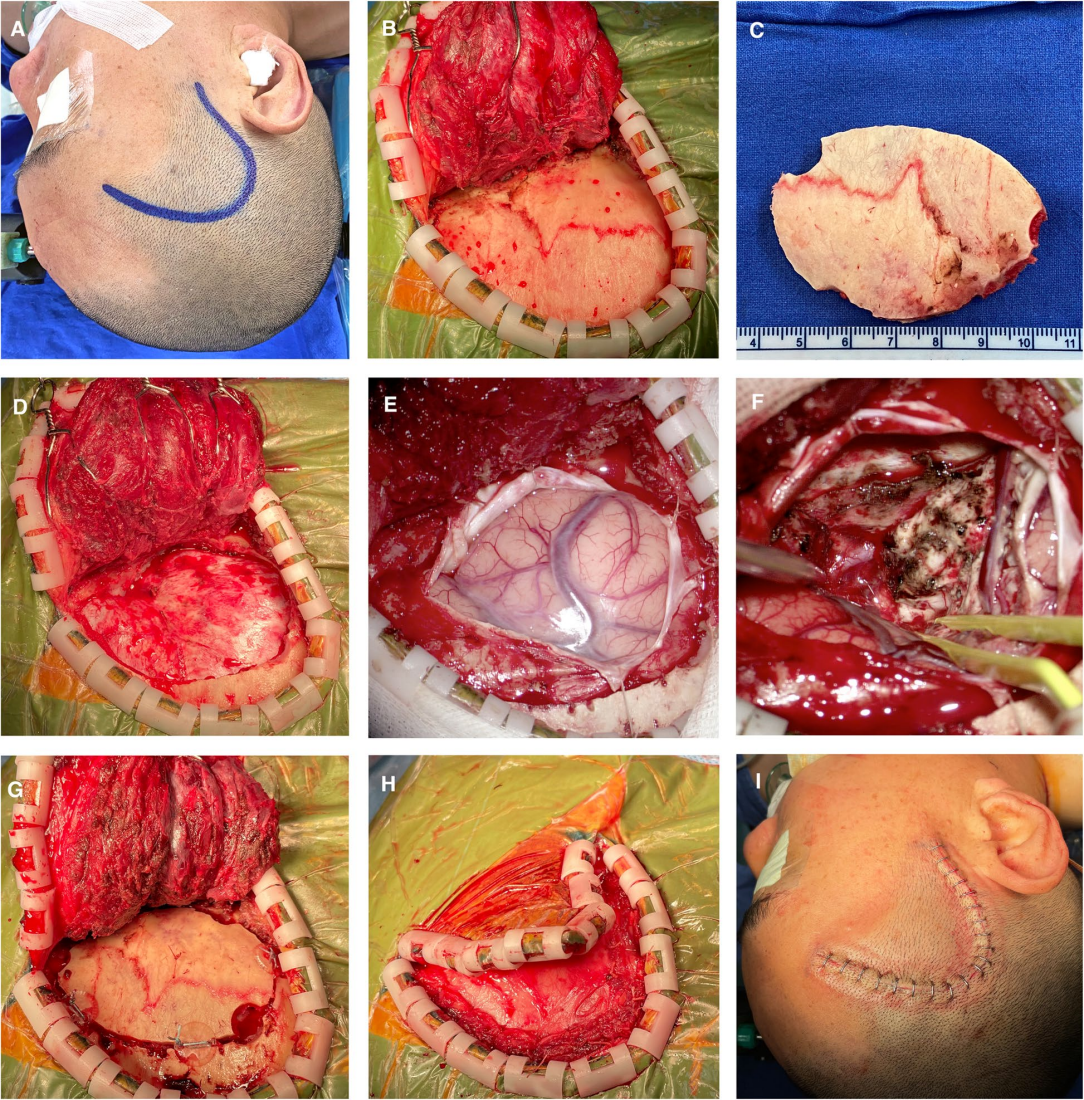
Craniotomy under the mini-temporal approach. **A** Patient is positioned in a supine position, with head rotated 45° towards the opposite shoulder, and a curved incision is made within the hairline of the temporal region. **B** Hook-shaped retractors are used to maximize bony exposure. **C** A small bone window is opened. **D** Opening of the bone flap. **E** Cutting open the dura and exposing the cortical areas as needed. **F** Removal of the temporal lobe lesion. **G** Complete restoration of the cranium. **H** Complete restoration of the temporal muscles. **I** Use a skin stapler to close the incision

The patient is positioned in a supine position, with head rotated 45° towards the opposite shoulder, vertex tilted downwards to the floor. A curved incision is usually made in the temporal region (Fig. 1A). Based on preoperative navigation images, incisions can be customized and made as small as possible.

Retraction is very important since the exposure can be very small. Usually 2 to 3 hook-shaped retractors for the flap will be used (Fig. 1B). Drippings should be fixed tightly by the circulating nurses for sufficient retraction.

**Fig. 2:**
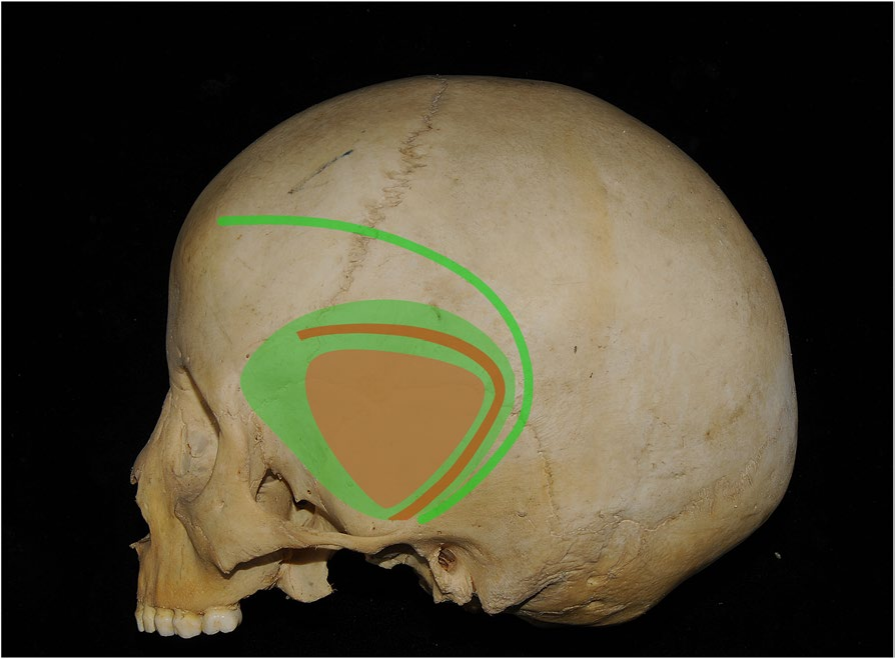
Comparison of incision and bone window size between the mini-temporal and classical pterional approaches. The orange line indicates the incision for the mini-temporal approach, the green line indicates the incision for the classical pterional approach, the blue area represents the bone window of the mini-temporal approach, and the yellow area represents the bone window of the classical pterional approach

The bony exposures are mainly the temporal bone and the greater wing of the sphenoidal bone. The extents are as follows: downwards, zygomatic arch root; upwards, 1 cm above the squamous suture; anterior, anterior extremity towards sphenoidal ridge after retraction (usually have a distance to the “keyhole”); and posterior, level of the external acoustic meatus (or more posterior according to position of the lesions). The classical craniotomy landmark, the keyhole, can seldom be reached in this approach. The first drill is usually at the root of the zygomatic arch. The second one, if necessary, is usually at the anterior and upper limit toward the direction of the keyhole. A small bone window is opened (Fig. 1C). The craniotomies extent usually equals the exposure after the flap retraction; usually, no extra bone should be removed after the bone flap removal (Fig. 1D). For a trained doctor, the procedure usually takes about 20 min and greatly reduces the size of the bone window (Fig. 2). The smaller bone window speeds up the craniotomy process and reduces the possibility of nerve damage compared to the traditional pterional approach.

After dura opening, the anterior extremity of the temporal lobe is hidden (Fig. 1E). The sylvian fissure is usually sufficiently exposed with minimal exposure of the inferior frontal gyrus. With adjustment of the microscope angle, elevation of the patient’s upper half, and with gradual lowering of the cerebral pressure during resection, the whole temporal lobe can be resected sufficiently and safely (Fig. 1F). Without excess cortical exposure, the cortex can also be effectively protected from accidental intraoperative injury. Excessive normal cortical exposure is also one of the shortcomings of the classical approach. Dura closure can be performed easily, and complete restoration of the cranium can be done (Fig. 1G). The temporal muscle can be completely restored and completely covers the bone window (Fig. 1H). Because there is no muscle separated from the temporal line and the temporal branch of the facial nerve is not affected, the muscle atrophy occurring in some of the traditional pterional approach will not occur in this approach. Finally, skin incisions were closed with a skin stapler (Fig. 1I). Because the incision is small, some incisions can even be sutured subcutaneously, which is not only neat but also can reduce the patient's hospital stay.

## Results

### Case illustrations

**Fig. 3:**
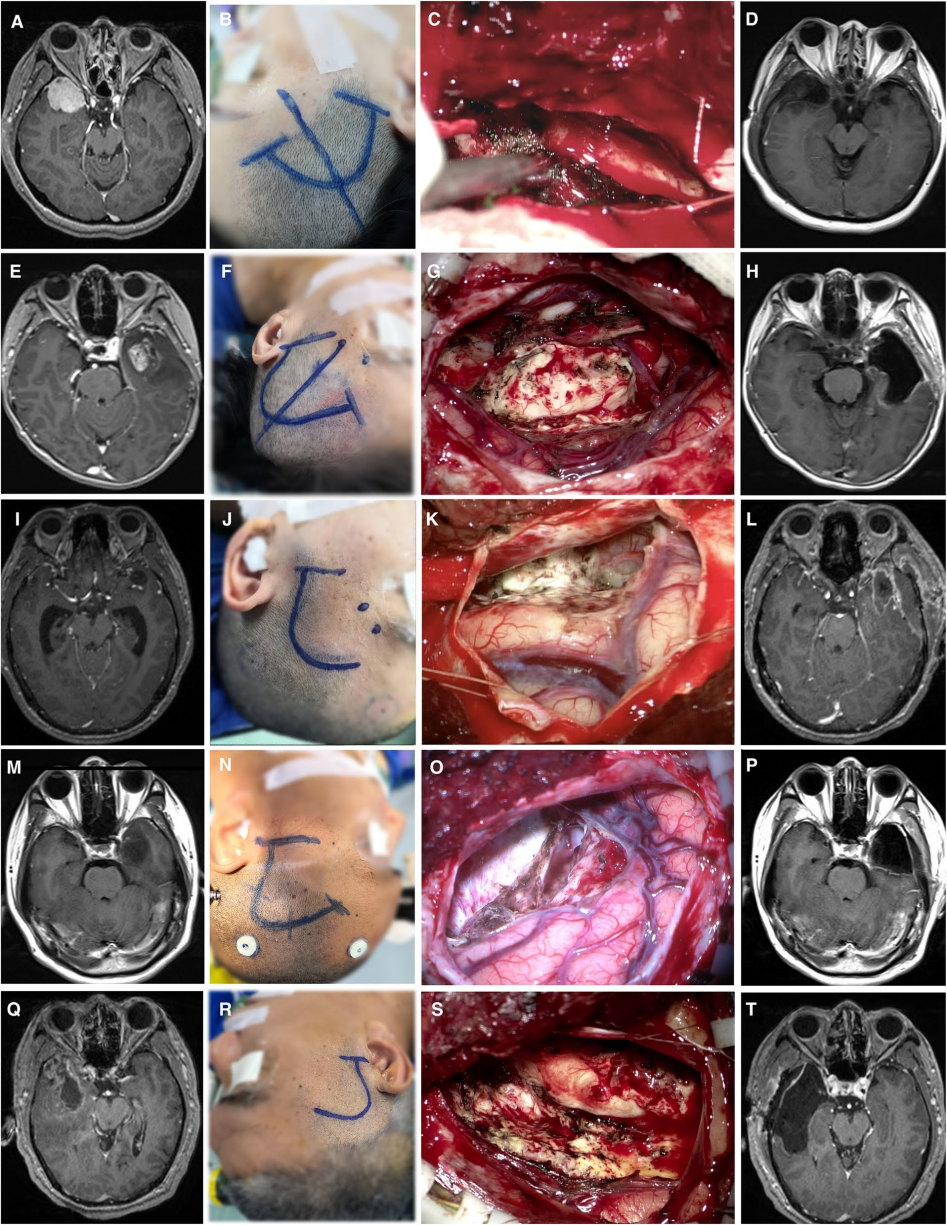
Illustration of the mini-temporal approach. As applied in a variety of tumor resections or open biopsy procedures, including meningiomas (**A**), gliomas (**E**, **M**, and **Q**), and chordomas (**I**). The incisions were individually designed according to the tumor size and location and the patient’s hairline (**B**, **F**, **J**, **N**, **R**). With a smaller incision and a bone window, most temporal lesions can be successfully removed (**C**, **G**, **K**, **O**, **S**), and MRI imaging can confirm total resection (**D**, **H**, **L**, **P**, **T**)

Informed consent was obtained from all individual participants. Patients consented to the publication of their images. The first patient was a 50-year-old woman who suffered seizure attack once. Magnetic resonance imaging (MRI) revealed a significantly enhanced mass in the right temporal lobe (Fig. 3A). We used the mini-temporal approach to access the tumor and perform resection of the tumor. The tumor is confirmed to be meningioma during surgery, and it is totally resected by Simpson grade I (Fig. 3 B and C). Postoperative MRI showed complete resection of the tumor, and postoperative pathology showed meningioma of WHO grade I (Fig. 3D).

The second patient was a 61-year-old female who suffered intermittent headache for 6 months. An enhanced lesion can be seen in the right temporal region (Fig. 3E). The mini-temporal approach was designed for performing “en bloc” resection of the tumor (Fig. 3 F and G). We performed anterior temporal lobectomy including the whole tumor, sparing the hippocampus. Post-op MRI confirmed the resection extent, and postoperative pathology showed a glioblastoma of WHO grade IV (Fig. 3H).

The third patient, who had numbness in the left side of the face for 4 months, showed multiple non-enhanced small lesions in the brain including left anterior Sylvian fissure region (Fig. 3I). We chose the mini-temporal approach to perform biopsy; the incision was made as close to the hairline as possible to approach the Sylvian fissure (Fig. 3 J and K). The lesion was removed completely, and postoperative pathology showed chordoma (Fig. 3L).

The fourth patient had blurred vision on the right eye. MRI showed a non-enhanced lesion in the left temporal lobe (Fig. 3M). The mini-temporal craniotomy was designed slightly larger, and “en bloc” resection of the tumor and anterior temporal lobe was performed. Post-op MR confirmed the resection extent (Fig. 3 N and O). Postoperative pathology revealed a astrocytoma of WHO grade II (Fig. 3P).

The last patient was a 59-year-old male who suffered headache for half a month. An enhanced lesion can be seen in the right temporal region (Fig. 3Q). The mini-temporal approach was designed for performing “en bloc” resection of the anterior temporal lobe as well as the whole tumor, sparing the hippocampus (Fig. 3 R and S). The lesion was removed completely, and postoperative pathology showed a glioblastoma of WHO grade IV (Fig. 3T).

In the above typical case, we designed minimally invasive incisions with the assistance of navigation and completed the operation through a minimally invasive incision with only adjusting the microscope angle. The smaller incision of the mini-temporal approach did not affect the microsurgical procedure and tumor resection.

## Discussion

In 1944, Dandy introduced the frontolateral craniotomy for the treatment of anterior circulation aneurysms, forming the basis of the classical pterional craniotomy. Kempe, Shepard, and Swain further developed this procedure and was greatly improved and popularized by Yasargil. Because of its versatility, this approach is widely used for approaching lesions in the circulation circle of Willis and the parasellar regions as well as the frontal and temporal lobes. Although brain retraction is a commonly used technique in neurosurgery, it leads to postoperative complications; the main complication is facial paralysis caused by damage to the temporal branch of the facial nerve, which is manifested by the disappearance of forehead wrinkles and eyebrow drooping; at the same time, nerve damage leads to muscle atrophy [2]. In a study evaluating masticatory function and facial nerve recovery after pterional approach surgery, 37.5% of patients had difficulty chewing hard food after surgery, and 25% of patients still had pain when chewing hard food 1 year after surgery [3]. Pterional craniotomy requires complete dissection of the temporal muscles and often unnecessarily exposes large areas of the cortex. The damage to normal tissues during surgery may seriously affect the daily life and social life of patients after surgery [3]. In the minimally invasive, personalized, patient-centered treatment process, we need to consider procedures that can reduce postoperative complications.

New surgical techniques have been proposed to reduce the exposure of pterional craniotomy, reduce hospitalization and operative times, reduce tissue trauma, create a comfortable postoperative period, lower costs, and improve esthetic and functional outcomes [4]. Figueiredo et al. confirmed that the surgical exposure area and the approach angles gradually increased with the progression of the Sylvian fissure dissection toward the pars triangularis at the level of the anterior ascending ramus, and further dissection did not increase the exposure [5]. Keyhole surgery is also a way to minimize damage to normal tissue. Keyhole approaches commonly used as alternatives to pterional approach include superciliary keyhole approach, supraorbital keyhole approach, and mini-pterional keyhole approach. The study has shown that the incidence of postoperative temporal atrophy was significantly lower in the mini-pterional group than in the pterional group during aneurysm surgery [6]. Despite facial wounds, the superciliary keyhole approach provided a higher level of patient satisfaction than the pterional approach [7]. Compared with the pterional approach group, the operative time and intraoperative blood loss in the supraorbital keyhole approach group were significantly shortened [8]. These keyhole approaches are suitable for tumor located at the anterior cranial fossa without significant temporal extension. Keyhole approaches mentioned above are mainly applicable to extracerebral lesions and meningiomas, while endoscopic operation or endoscopic assistance is often required for intracerebral lesions. In mini-temporal craniotomy, the temporal lobe exposure will be larger than that of approached above to better expose both the temporal lobe and the Sylvian fissure region when approaching the arterial circle of Willis, which makes the en bloc resection of the temporal lobe tumors possible; however, the temporal lobe and middle cranial fossa tumor cannot be fully exposed by the above keyhole incision.

To protect arteries and nerves in the temporal region, it is necessary to dissect the muscle and periosteum together during the separation of the temporalis. Before surgeons began to intentionally protect the temporal branch of the facial nerve during craniotomy, frontalis muscle palsy occurred in 30% of patients who underwent scalp flap subgaleal elevation with separate temporalis separately elevation [9]. The loose areolar tissue between the galea and the superficial layer of temporal fascia is where the temporal branches to the frontalis muscle run. Thus, the interfascial-subpericranial and subfascial-subpericranial techniques can retain innervation of the frontalis muscle [10]. The hairline at the temple is also comsidered a marker, and the area behind it is safe to dissect [10]. In the mini-temporal incision design, the frontal end of the incision never surpasses the hairline at the level of temporal line, and a one-layer skin-galea-muscle flap is detached from the cranium, effectively avoiding the injuries of facial nerve. Because the mini-temporal incision is usually small, the surgical bone window is completely located underneath the temporalis muscle, allowing it to be completely repositioned postoperatively. The cranium is usually milled in one piece with no extra bone removal, and the cranium can be restored intactly. Mini-temporal craniotomy does not extend to the frontal side of the skull, so the frontal sinus will not be invaded, reducing the possibility of postoperative cerebrospinal fluid leakage and infection. Mini-temporal incisions require less hair to be shaved, so fewer holes are required to be drilled and fewer temporalis to be separated.

With the promotion of the concept of minimally invasive surgery, the protection of brain tissue in the surgical field has become a consensus, but the protection of other tissues along the surgical path should be further promoted. Long-term postoperative pain during mastication may lead to a a dental professional than a neurosurgeon. In addition, patients may use hair to shield the atrophy of the temporalis muscle, believing that atrophy and pain are a necessary price to pay for a successful surgery. Recent articles point out that while traditional pterional may lead to a range of cosmetic and functional problems, few studies have focused on cosmetic outcomes and patient satisfaction; only 10 studies (26.31%) involved patient satisfaction with esthetics, and only 7 studies (18.42%) reported cosmetic outcomes as the primary outcome [2]. Even the questionnaire-based postoperative assessment for cosmetic outcomes and patient satisfaction was just recently discussed in an article [3]. Therefore, minimally invasive approaches like the mini-temporal approach have become very important. According to the previous surgical experience of our center, mini-temporal approach was used in tumor resection of temporal area, which greatly reduced the impact of normal skin, muscle, bone, and cortex during craniotomy and limited the surgical trauma to the tumor area, truly reflecting the minimally invasive concept.

Naturally, mini-temporal apporach has limitations. First, bone window size is limited, and it requires the surgeons to adjust the microscope to find the best angles. Second, when deep bony drilling in the surgical field is required, when brain edema or hematoma high intracranial occurs, or when fully dissection of the sylvian fissure is required for aneurysms, the mini-temporal approach does not provide the same convenience and safety as the classical pterional approach. Therefore, the choice between mini-temporal and classical pterional approaches should be based on the anatomical positions and clinical/ radiological features of the lesions.

## Conlusion

Mini-temporal approach can effectively shorten the time of craniotomy and closure, decrease the size of bony removal and the chance of nerve injury, and facilitate the restoration of temporalis, thus improving cosmetic and functional outcomes and reduces the risk of unintentional cortical injury. This approach fully demostrates the minimally invasive principle in neurosurgery.

## Data Availability

This article is available as a preprint at the following web site: https://www.researchgate.net/publication/352348718_Mini-temporal_approach_as_an_alternative_to_the_classical_pterional_approach_for_resective_temporal_region_surgeries_Technical_note. However, the content of this article is not completely consistent with the preprint and has been modified to some extent.
